# Dietary Patterns among Older People and the Associations with Social Environment and Individual Factors in Taiwan: A Multilevel Analysis

**DOI:** 10.3390/ijerph19073982

**Published:** 2022-03-27

**Authors:** Yi-Hsuan Lin, Hui-Chuan Hsu, Chyi-Huey Bai, Wen-Chi Wu

**Affiliations:** 1 School of Public Health, Taipei Medical University, Taipei 11031, Taiwan; m508108003@tmu.edu.tw (Y.-H.L.); baich@tmu.edu.tw (C.-H.B.); 2 Research Center of Health Equity, College of Public Health, Taipei Medical University, Taiwan 11031, Taiwan; 3 Department of Public Health, College of Medicine, Taipei Medical University, Taipei 11031, Taiwan; 4 Department of Health Promotion and Health Education, School of Education, National Taiwan Normal University, Taipei 10610, Taiwan; wenchiwu@ntnu.edu.tw

**Keywords:** dietary behavior, dietary belief, social environment, age-friendly city, older adults, health promotion

## Abstract

Individual factors relating to dietary behaviors are widely explored. However, the effects of social environment on dietary patterns for the older people are less explored. The purpose of this study was to identify dietary patterns among older people in Taiwan and to examine the relationship of dietary patterns with social environment and individual factors. The current study used the 2013–2016 Nutrition and Health Survey in Taiwan. The sample was representative at the national and city levels. Only those who were aged 55 years old and above were included for analysis (*n* = 2922); the mean age of the participants was 68.62 (SD = 8.76). The city-level data, including population characteristics, food availability, and age-friendly city indicators, were obtained from the open data and survey report of government. Three dietary patterns were identified: high protein-vegetable (41.6%), high sweets and low protein-vegetables (37.9%), and high viscera and fats (20.5%). The results of multilevel multinomial logistic regressions showed that marital status, economic status, education, drinking alcohol, dietary belief, living a the city with more food availability, and bus accessibility were related to dietary patterns. Dietary patterns are related to the individual-level factors and social environment. Healthy dietary beliefs and age-friendly environments are beneficial to promoting healthy dietary patterns.

## 1. Introduction

Dietary intake is related to chronic disease, frailty, cognitive function, and physical function in older people [1]. Previous studies have explored the dietary patterns of individuals but are less focused on older people, and placed less emphasis on environmental constraints or barriers to food intake, especially for older people. The purpose of this study was to identify the dietary patterns of middle-aged and older people in Taiwan and to explore the individual and environmental factors related to dietary patterns.

The identification of dietary patterns can be determined a priori by the dietary index or scores, such as the Health Eating Index (HEI) [2], or the patterns can be identified based on the dietary records of food intake a posteriori [3]. The latter is more appropriate for describing dietary patterns in a comprehensive way and is not confined to food culture.

Dietary patterns can vary and depend on the individual’s lifestyle and culture. For example, there are three common French dietary patterns: healthy diet, Western diet, and traditional diet [4], while another French study identified five dietary patterns: traditional, prudent, diversified, processed, and sandwiches [5]. The USA has five dietary patterns: convenience, plant-based, sweets/fats, southern diet, and salad with wine [6]. One study from Rotterdam defined dietary patterns using the Dutch Healthy Diet Index and suggested three kinds of dietary patterns: traditional, carnivore, and health conscious [7]. A study in Taiwan suggested two kinds of dietary patterns for middle-aged and older people: one pattern is more vegetables and fruits, and the other is more meat and processed food [8].

Individual factors related to dietary behaviors include demographics, health conditions, health-related behaviors, as well as dietary habits and belief. Individuals who are older and female are more likely to have a healthier dietary pattern [4,9]. Lower education is related to lower intake of fruits and vegetables and higher consumption of fast foods, and higher education is related to better dietary quality [10]. Higher socioeconomic status is usually related to better health literacy and dietary knowledge [11], but there is research indicating that higher income may be related to lower intake of vegetables, fruits, and fish and higher intake of meats in different places [12]. Older people with smaller social networks or fewer social contacts generally have a lower quality diet and tend to have similar food intake [13]. The composition of households also affects the choice of vegetables and fruits [14]. Dietary behaviors may contribute to different health outcomes, and people also need to adjust their dietary habits based on their health conditions [15]. Health-related behaviors may co-occur as a lifestyle; healthy dietary behavior usually co-occurs with other behaviors to form a healthier lifestyle [16].

Environmental factors can be divided into population composition, food availability, and age-friendly city environment. Demographic composition, culture, and value of neighborhoods can shape dietary habits [17]. Older people living in rural areas or areas with lower population density consume more vegetables and fruits [18] or a more traditional diet (vegetables and dairy) [4]. The socioeconomic status of the areas may be related to the residents’ ability to choose a healthy lifestyle, which involves better health literacy and economic status [19]. Relative deprivation may also stimulate an individual’s desire for food to compensate deprivation feeling and thus leading to obesity-related dietary behaviors [20]. Food availability in the environment also affects food intake. More exposure to fast food outlets leads to more fast-food consumption [10], but the city development and different sub-cultures also affect the food availability [19]. The income distribution of the living areas may be related to the availability of different food sources. The least deprived areas have the longest distance to these retailers [21]. An age-friendly physical environment is expected to involve easy access to food purchases. The accessibility of public transportation is related to food intake behaviors, especially in low-income neighborhoods [22,23].

Although factors affecting dietary behaviors have been explored and dietary patterns have been identified in different cultures, few studies have identified dietary patterns for older people and simultaneously examined both individual and environmental factors from an age-friendly city perspective for older people. The purpose of this study was to identify the dietary patterns of middle-aged and older people in Taiwan and to explore the individual and environmental factors related to dietary patterns. The research hypotheses are: (1) an individual’s dietary belief is related to his/her dietary patterns. (2) City factors, in terms of city and population characteristics, food availability, and age-friendly city characteristics, are related to an individual’s dietary pattern.

## 2. Materials and Methods

### 2.1. Data and Sample

This study was a cross-sectional study using secondary data. The individual data were obtained from the Nutrition and Health Survey in Taiwan (NAHSIT) 2013–2016 data. The NAHSIT is a source of nationally representative data in Taiwan. A three-stage probability proportional to the size sampling plan was performed in 160 primary sampling units that cover 20 cities and 359 townships or city districts. The sample was representative at the national and city levels. The participants in different cities were interviewed in turn from 2013 to 2015. The questionnaires were conducted via face-to-face interviews. Participants who had Alzheimer’s disease or severe health conditions (such as Alzheimer’s disease or Parkinson’s disease), pregnant or breast feeding, who had hearing and visual impairments, who were living in institutions, or unable to communicate, and who were living in institutions were excluded [24]. In this study, only those who were aged 55 years old and above were included for analysis. The analysis sample included 2922 individuals.

The city-level data were obtained from the open data of the government, including the 2016 Population Statistics [25], the 2016 Survey of Family Income & Expenditure [26], the 2016 Industry commerce and Service Census [27], and the 2013–2016 Age-Friendly Environment Monitor Study of the Health Promotion Administration [28].

### 2.2. Measures

The dependent variable was a dietary pattern, which was measured by the Food Frequency Questionnaire [29], a common tool to measure the dietary intake. The frequency of food intake over a week was assessed. Dietary patterns were identified by the Food Intake Frequency Questionnaire for 48 food items.

#### 2.2.1. Individual Factors

Individual factors included demographic factors, health behaviors, health beliefs, chronic disease number, and self-rated health. Demographic characteristics included age, sex, education, working status (yes/no), marital status (having a spouse or not), household income, subjective economic status (insufficient, fair, or abundant), and living district in the city (urban or rural), according to the report of the National Development Council [30]. Health behaviors included smoking (yes/no), alcohol consumption (yes/no), betel nut chewing (yes/no), and dietary habits (vegetarian or nonvegetarian). Dietary beliefs represent personal attitudes and knowledge about specific categories of food, including fried food, whole grains, dairy, fruits, and vegetables (please see Supplementary Table S1). The dietary belief questions were only provided to participants less than 65 years old. The score of each item ranged from 1 (strongly disagree) to 5 points (strongly agree). All reverse questions were recoded to calculate scores. A higher score indicated a more acceptable attitude toward the types of food. The disease number was the summed number of morbidities of 29 chronic diseases. Self-rated health status was measured by the following question: “How is your health status compared with others at your age/sex?” The score ranged from 1 (much worse) to 5 (much better), when compared to others.

#### 2.2.2. City Factors

We categorized city-level factors into three dimensions: city and population characteristics, food availability, and age-friendly city characteristics. The indicators of the city and population characteristics included the population density (defined as the average population per square kilometer), the size of the older population (who were 65 years old and older), the size of the population to have undergone higher education, the median household income, the household income distribution (Gini coefficient), and the park green area per 10,000 people. The Gini coefficient measures the inequality among values of household income distribution, ranging from 0 to 1; a higher value indicates a more unequal distribution. Food availability indicators included density of convenient stores, density of vegetable and fruit stores, density of meat sores, density of seafood stores, density of other food stores, density of restaurants and diners. The data of age-friendly city characteristics^28^ included four barrier-free environment indicators: barrier-free sidewalk percentage, barrier-free pathway outside of residence, barrier-free bus percentage, and the percentage of bus accessibility (accessible within 500 m from home).

### 2.3. Ethics

The study was conducted according to the guidelines of the Declaration of Helsinki and approved by the Institutional Review Board.

### 2.4. Analysis

Dietary patterns were analyzed by factor analysis and cluster analysis. First, the 48 food items were analyzed by factor analysis and extracted into 8 factors, i.e., animal protein (meat, fish, eggs); whole grains/fruits/dairy/nuts; vegetables; beans, melons, and pickles; viscera and blood; cookies and cakes; ice cream and fast food; and milk powder (please see Supplementary Table S2). The individual’s food intake by factors analysis was then defined. Next, the dietary patterns of the participants were categorized by cluster analysis of the food intake of factor scores of factor analysis. Hierarchical cluster analysis was conducted first to explore the best cluster numbers, and then k-means cluster analysis was used to define the dietary pattern groups.

Multilevel multinomial logistic regression was used to estimate the individual- and city-level effects with random effects on dietary patterns. The multilevel model can deal with data structures with clustered/nested characteristics, such as individual-level data nested within city-level data. In this study, the outcome variable (dietary pattern) was a multiple-categorical variable. In this study, the individual factors were the level 1 variables, and the city factors were the level 2 variables. The random effect of the model was assumed to be the individual factors nested in cities [31]. The analyses were conducted by SPSS Statistics 22 software (IBM, SPSS Inc., Chicago, IL, USA) by using generalized linear mixed models.

## 3. Results

The distributions of study participant characteristics are shown in Table 1. Three dietary patterns were identified by cluster analysis (please see Table 2). The high protein and high vegetable pattern (41.6%) was characterized by higher intake of animal protein and vegetables; the high sweet and low vegetable and protein pattern (37.9%) consisted of more intake of cookies and cakes but less animal protein and vegetables; and the high viscera and fat pattern (20.5%) was characterized by the intake of more viscera, blood, and fats and less vegetables.

The results of the multivariate analysis of dietary patterns and individual and city factors for middle-aged (age 55–64) and older people (age 65+) are presented in Table 3. The reference group of the dependent variable was the high protein and vegetable pattern. Individual factors and city factors were examined for their relationship with dietary patterns using bivariate analysis. In Table 3 of the middle-aged participants, Model 1 included both individual factors and city factors, except for the dietary belief variables. In Model 1, compared with the participants with the high protein-vegetable pattern, the participants with the high sweets and low protein-vegetable pattern were more likely to have lower education status (OR = 2.954 for the illiterate group); city-level factors were not significant. Participants performing a high viscera and fats pattern were more likely to be working (OR = 1.439) and live in cities with more equal income (OR = 0.838 for income inequality).

When incorporating dietary beliefs in Model 2 for the middle-aged participants, compared with the high protein-vegetable pattern group, the participants of the high sweets and low protein and vegetable pattern were more likely to have no spouse (OR = 0.442), have lower economic status (OR = 0.446 for the abundant group), have lower vegetable–fruit beliefs (OR = 0.336), and more likely to live in cities with a lower percentage of bus accessibility (OR = 0.971). At the same time, when including dietary beliefs in the model, personal education, working status, and city income distribution were no longer significantly related to the high viscera–fat dietary pattern.

Model 3 shows the results for people who were 65 years and above. Compared with participants with the high protein–vegetable pattern, individuals in the high sweets and low protein–vegetable groups were more likely to have no spouse (OR = 0.555), lower education (OR = 3.317 and 1.93 for illiterate and elementary school, respectively), and poorer economic status (OR = 0.418 for the abundant group and OR = 0.535 for the fair group) and living in the cities with more seafood stores (OR = 1.064). At the same time, participants with a high viscera and fat pattern were more likely to have lower economic status (OR = 0.588 for the abundant group and 0.714 for the fair group), drink alcohol (OR = 2.202); the city factors were not significant.

## 4. Discussion

This study identified the dietary patterns for middle-aged and older people in Taiwan and examined the individual-level and city-level factors related to dietary patterns using multilevel analysis with a nationally representative sample. In addition to dietary beliefs and individual factors, food availability and an age-friendly environment were also related to dietary patterns.

In general, Taiwanese older people’s dietary patterns are different from patterns in other countries. This study identified a healthier dietary pattern, the high protein-vegetable pattern, was similar to the healthy diet group in France [4]. However, the combination of food kinds in Taiwan was different from other countries; other countries tend to separate higher meat and higher vegetables into two different diets [4,5,6,7,8]. In addition, viscera and blood products are commonly found in Taiwan’s traditional diet, but not necessarily acceptable in some Western diets. Dairy products are often acceptable in Western dietary culture, but older people in Taiwan do not often report intake of milk or dairy products. Older people in Taiwan also have less intake of convenient foods or sandwiches than their counterparts from Western countries. In our study, a high sweet and low protein–vegetable diet and high viscera–fats diet were identified, but the sweet-and-fat diet was grouped together in the U.S. study [6].

Older people with lower education and lower economic status showed a consistent effect in that they were more likely to have a high sweet and low protein–vegetable diet, similar to previous research studies [4,5,6,10]. Socioeconomic status and education represent their health literacy and the ability to purchase healthy food.

Dietary belief affects dietary behaviors, since middle-aged participants who had higher vegetable and fruit beliefs were less likely to have high sweet and low protein–vegetable patterns. When including dietary belief, education, working status, and city’s income distribution did become not significant. These results imply that changing older people’s dietary beliefs would be the key to changing their dietary behaviors, even to reduce the socioeconomic effects on dietary behaviors.

Previous studies have found the clustering of healthy behaviors [16]. In this study, the older high viscera–fat pattern participants were more likely to drink alcohol than the high protein–vegetable group. It seems that the older people with the high viscera–fat pattern had higher probabilities of conducting unhealthy behaviors (drinking alcohol and chewing betel nuts). The high viscera–fat pattern group may be at high risk for co-occurring unhealthy behaviors.

Previous studies showed that having more social contacts or social support was related to healthier dietary behavior [13,14]. The middle-aged and older people who had a spouse were more likely to consume a high protein–vegetable diet but less likely to consume a high sweet and low vegetable–protein diet. When older adults have a spouse, it is more likely that they cook and eat together, and thus the food intake will be more nutrient-balanced.

Living in a city with lower household income was expected to be related in the development of a high-sweets-and-low-vegetable-and-protein pattern and high-viscera-and-fat pattern. However, the variable was not significant, probably because individuals’ economic status is stronger than the city’s economic status. The Gini coefficient was not significantly related to dietary patterns when including individual factors. It is possible that the Gini coefficients of all the cities were very close.

Food availability has been found to be related to dietary behaviors [11]. However, in this study, older adults who lived in cities with a higher density of seafood stores were more likely to eat a low protein–vegetable diet. The possible reason is that older people are more likely to be of lower socioeconomic status; purchasing seafood or other protein is more expensive for them, especially in the cities with more fishing or other primary industries.

Living in cities with lower bus accessibility was more likely to result in a high sweet and low protein–vegetable diet pattern. In order to consume a diet with more protein and vegetables, fresh foods must be purchased very often. Accessible transportation would make it easier for older, frail, or disabled people to go out and shop for groceries [22,23]. An age-friendly city infrastructure may help older people to live a healthy lifestyle. The older people in Taiwan enjoy the social welfare of a bus subsidy. However, bus accessibility was not significantly related to dietary patterns for older people. It is possible that older people may go shopping for food nearby but are less likely to travel to buy food by bus; shopping is the errand that is often conducted by the younger members in the family.

There are some limitations to this study. First, the data were secondary data obtained from part of the NAHSIT data; some variables were unavailable. In addition, the questionnaires were different by age group. Health belief questions were not asked for older people who were 65 years old or more. Second, the area-level data were retrieved from the open data of the government. Only the nearest year data were applied. In addition, most of the area-level data were released at the city level but not in a smaller unit. There are variations within a city. Third, the data were cross-sectional and, thus, the causal relationships of individual- and city-level factors with dietary patterns cannot be confirmed.

## 5. Conclusions

We identified three dietary patterns for middle-aged and older adults in Taiwan. The healthier (high protein–vegetable) dietary pattern was related to dietary beliefs, higher socioeconomic status, and marital status, and the other two less healthy dietary patterns were related to socioeconomic status, food availability, and limitations of a barrier-free environment. The identified dietary patterns can be used to measure health behaviors of older people, and the health outcomes of dietary patterns of older people should be monitored in the future research. Healthy dietary belief can be improved through health education in dietary knowledge and food choice, thus health education in healthy diet would be beneficial to build up healthy dietary patterns. Age-friendly environment affects the food accessibility and availability, particularly for frail older adults. Policy makers should endeavor to promote dietary literacy through health education and build age-friendly cities where there are less barriers to obtaining healthy food. Longitudinal research on environmental factors and dietary patterns is also suggested.

## Figures and Tables

**Table 1:** The description of the sample demographics, health-related behaviors, health conditions, and dietary belief.

Variables	*n*	Mean (SD) or %
Age	2954	68.62 (8.76)
55–64	1034	35.0%
65–74	1151	39.0%
75 and above	769	26.0%
Sex		
Male	1477	50.0%
Female	1477	50.0%
Education		
Illiterate	316	10.7%
Elementary school or non-formal education	1211	41.0%
Junior high school	409	13.8%
Senior high school	519	17.6%
College/University or above	499	16.9%
Marital status		
No spouse	829	28.1%
Having spouse	2125	71.9%
Working status		
Yes	866	29.4%
No	2084	70.6%
Subjective economic status		
Abundant	407	14.0%
Fair	1613	55.7%
Insufficient	878	30.3%
Districts		
Urban	2275	77.0%
Rural	679	23.0%
Smoking		
No	1934	67.1%
Yes	949	32.9%
Drinking alcohol		
No	1544	53.6%
Yes	1338	46.4%
Chewing betel net		
No	2464	85.6%
Yes	416	14.4%
Self-rated health (1~5)	2911	3.10 (1.05)
Chronic disease numbers	2954	2.25 (1.89)
Dietary habit		
Non-vegetarian	2777	94.0%
Vegetarian	177	6.0%
Dietary belief in average (1~5)		
Vegetables and fruits	897	3.66 (0.34)
Milk and dairy	874	3.46 (0.47)
Whole grains	802	3.34 (0.46)
Fried food	896	3.78 (0.36)

Note: *n* = 2594. Missing cases were excluded from the analysis.

**Table 2:** Dietary patterns of older people identified by cluster analysis.

Food Categories	Dietary Patterns		
	Cluster 1: High Protein and High Vegetables (*n* = 1215, 41.6%)	Cluster 2: High Sweets and Low Vegetable and Protein (*n* = 1108, 37.9%)	Cluster 3: High Viscera and Fats (*n* = 599, 20.5%)
Animal protein	0.42959	−0.63664	0.30625
Whole grains, fruits, and dairy	0.01901	−0.14853	0.23618
Vegetables	0.37722	−0.41328	−0.00068
Viscera and fats	−0.49281	−0.30736	1.56814
Melon and bamboo	0.32363	−0.38685	0.05913
Ice cream and fast food	−0.04850	−0.01795	0.13157
Sweets	−0.25075	0.24662	0.05243
Pickles and others	−0.29625	0.30795	0.03126

**Table 3:** Dietary patterns and related individual and city factors for middle-aged (age 55–64) and older people (age 65+) by multi-level multinomial logistic regression (odds ratios).

Variables	Model 1: Middle-Aged Participants (Age 55–64) with Individual and City Factors (without Dietary Belief) (*n* = 987)		Model 2: Middle-Aged Participants (Age 55–64) with Individual and City Factors (with Dietary Belief) (*n* = 952)		Model 3: Older Participants (Age 65+) with Individual and City Factors (without Dietary Belief) (*n* = 1769)	
	High Sweets and Low Protein and Vegetables	High Viscera and Fats	High Sweets and Low Protein and Vegetables	High Viscera and Fats	High Sweets and Low Protein and Vegetables	High Viscera and Fats
Individual factors						
Age	0.947	0.966	0.942	0.986	1.007	0.977
Sex (male)	0.712	0.882	0.710	0.691	0.907	0.890
Work (yes)	1.088	1.439 *	0.987	1.482	1.021	1.244
Marital status (having spouse)	0.495 **	0.814	0.442 **	0.735	0.555 ***	0.782
Education						
Illiterate	2.954 *	0.276	2.278	0.594	3.317 ***	1.273
Elementary school	1.324	0.896	1.144	0.900	1.938 ***	0.778
Primary high school	1.134	0.813	0.930	0.647	1.422	0.900
Subjective economic status						
Abundant	0.578 *	1.514	0.446 *	1.641	0.418 ***	0.588 *
Fair	0.787	1.334	0.689	1.176	0.537 ***	0.714 *
Drinking alcohol (yes)	0.901	1.169	1.025	1.337	1.209	2.202 ***
Smoking (yes)	1.104	1.346	0.865	1.697	0.927	0.972
Chewing betel nut (yes)	1.220	1.262	1.301	0.862	1.030	1.305
Self-rated health	0.936	0.969	1.031	1.012	0.926	1.011
District (urban)	0.733	0.717	0.538	0.703	1.176	1.448
Dietary health belief						
Fried food			0.964	0.595		
Whole grains			1.422	0.759		
Vegetables and fruits			0.336 **	0.913		
Dairy			0.774	1.248		
City factors						
Population density	0.848	0.915	0.839	0.906	1.014	1.042
Older people percentage	1.195	1.000	1.248	0.960	1.026	0.933
Income inequality distribution	0.882	0.838 *	0.866	0.867	0.948	0.997
Median household income	1.022	1.010	1.015	1.021	1.003	0.989
Convenient store density	1.218	0.986	1.393	0.761	0.947	1.040
Seafood store density	1.033	1.004	1.064	1.045	1.064 *	1.032
Other food store density	1.019	1.022	1.005	1.012	1.017	0.988
Barrier-free pathway	0.990	0.992	0.979	0.986	1.008	1.007
Accessibility to bus stop	0.981	0.988	0.971 *	0.984	0.995	1.003
Random effect	0.102	0.103	0.176	0.176	0.053	0.100
Model fit	AIC = 7393.978		AIC = 5446.490		AIC = 13,662.981	
	BIC = 7403.655		BIC = 5455.456		BIC = 13,673.876	
	−2LL = 7389.965		−2LL = 5442.472		−2LL = 13,658.974	

Note: Analysis by multilevel multinomial logistic regression. The reference groups included: dietary pattern (high protein/vegetables), sex (female), working (no), marital status (no spouse), education (senior high school and above), perceived economic status (insufficient), district (rural), drinking alcohol (no), smoking (no), chewing betel nut (no); other variables were ordinal or continuous. AIC: Akaike Information Criterion; BIC: Bayesian information criterion; −2 LL: −2 log likelihood. * *p* < 0.05, ** *p* < 0.01, *** *p* < 0.001.

## Data Availability

We do not have the right to share the NAHSIT data.

## Supplementary Materials

The following supporting information can be downloaded at: https://www.mdpi.com/article/10.3390/ijerph19073982/s1, Table S1: Dietary Belief Questions; Table S2: Food categories by factor analysis.
