# Supplementary material for: Dietary Patterns among Older People and the Associations with Social Environment and Individual Factors in Taiwan: A Multilevel Analysis
Source: Int J Environ Res Public Health. 2022 Mar 27;19(7):3982. doi: 10.3390/ijerph19073982 (PMC8998054; doi:10.3390/ijerph19073982)
Supplement: Supplementary file 1 [file ijerph-19-03982-s001.zip › ijerph-1623360-supplementary.pdf]

Supplement

Table S1. Dietary Belief Questions

Categories of dietary belief	Questions (each item scored 1-5, indicating very disagree to very agree)
Vegetables and Fruits	1. To increase intake vegetables and fruits keep me healthy.<br>2. Eating more vegetables and fruits help to reduce weight or maintain ideal weight.<br>3. Eating more vegetables and fruits costs more money.<br>4. Not many kinds of vegetables and fruits that I like.<br>5. Eating more vegetables and fruits may avoid constipation.<br>6. Eating more vegetables and fruits may present some kinds of chronic diseases, such as cancer, hypertension, and cardiovascular diseases.<br>7. I worry eating vegetables and fruits may intake too much pesticides.<br>8. I feel trouble to increase intake of vegetables and fruits.<br>9. In general, increase eating vegetables and fruits is beneficial for me.<br>10. I don't often have my favorite vegetables and fruits at home.<br>11. When I eat outside, it's not easy to eat vegetables and fruits for me.<br>12. More intake of vegetables and fruits make my diet more balanced and nutritious.<br>13. More intake of vegetables and fruits help my skin and beauty.<br>14. To increase the intake amount of vegetables and fruits is very difficult for me.<br>15. Eating more vegetables and fruits make my body feel more comfortable.
Dairy food	1. I believe dairy is nutritious.<br>2. Intake of dairy make me feel uncomfortable, such as diarrhea, constipation, or allergy.<br>3. I don't favor most dairy food.<br>4. Eating dairy food may prevent osteoporosis.<br>5. Increasing intake of dairy food is good for me.<br>6. I believe intake of dairy food is bad for health.<br>7. Dairy food is a good choice for snack (such as milk, cheese, and yogurt).<br>8. I like to mix dairy food with other food (such as milk mixed with soup, cheese in the burger).<br>9. I don't often have dairy food at home.<br>10. To increase dairy food intake is very difficult for me.
Whole-grain food	1. Using whole grains to replace refined grains makes my diet more balanced and nutritious.<br>2. Eating more whole-grain food may prevent constipation.<br>3. Most whole-grain food is not easy to chew.<br>4. Eating more whole-grain food makes my body more comfortable.<br>5. In general, eating more whole-grain food is good for me.<br>6. I don't often have whole-grain food at home.<br>7. When I eat outside, I don't often have chances to eat whole-grain food.<br>8. To choose whole-grain food cost more money.<br>9. I am not sure what foods in my daily diet are whole-grained.

	10. To increase intake of whole-grain food is difficult to me.<br>11. To eat more whole-grain food may prevent chronic diseases, such as cancer, cardiovascular diseases, hyperlipidemia, etc.
Fried food	1. I feel hard to resist the attraction of fried food.<br>2. Eating less fried food may prevent me obesity.<br>3. Eating less fried food may reduce the possibility of vascular disease or strokes.<br>4. Eating less fried food makes me feel hungry and not satisfied.<br>5. I feel inconvenient to buy other food to avoid fried food.<br>6. Eating less fried food may prevent acnes.<br>7. Eating less fried food may prevent cancers.<br>8. If I buy other food to avoid fried food, it will cost me more.<br>9. When I eat with my family or friends, it is hard to avoid fried food.<br>10. I am not sure what foods in my daily diet are fried food.<br>11. To eat less fried food is very difficult to me.

Table S2. Food categories by factor analysis

Food items (times/week) \ Factor loadings	Factors (Food Categories)							
	1Animal protein	2Whole grain, fruits and dairy	3Vegetables	4Viscera and fats	5 Melon and bamboo	6 Ice cream and fried food	7Sweets	8 Pickle and others
Other seafood	**.590**	.192	.013	.158	.066	.070	.011	.122
Poultry	**.587**	-.032	.139	.225	.121	.016	.058	-.148
Shell fish	**.581**	.135	.075	.104	.149	.091	-.021	.091
Processed seafood	**.571**	-.015	.090	.010	-.035	.035	.125	.231
Fish with bones	**.527**	.196	.060	.071	.199	-.151	.031	.140
Deep-ocean fish	**.515**	.210	.150	-.021	.101	.096	-.085	-.073
Livestock lean	**.501**	-.076	.133	.158	.101	-.080	.073	-.216
Minced meat	**.492**	-.155	.112	.058	-.091	.149	.132	.128
Processed meat	**.455**	-.068	.047	.108	-.084	.128	.265	.184
Livestock half-fat	**.437**	-.019	-.025	.212	.030	.140	.034	.012
Seafood product	**.370**	.096	-.094	.004	.035	.143	.089	.239
Seasoned vegetable	**.310**	.138	.238	.094	.170	-.193	-.067	-.056
Seafood eggs	**.241**	.192	.012	.191	-.103	.086	.158	-.002
Whole grain bread	.087	**.599**	.070	.012	.028	.129	.026	-.010
Whole grain rice	-.112	**.568**	.170	.002	.106	-.022	.029	-.074
Nuts	.033	**.560**	.144	.138	.077	-.008	.149	.069
Dried fruits	.051	**.555**	.127	.109	.004	-.002	.160	.101
Cheese	.049	**.491**	.068	.065	-.033	.249	.086	.070
Fresh fruits	.200	**.445**	.216	-.149	.282	-.012	.123	-.198
Jam	.134	**.309**	-.074	-.027	-.036	.079	.002	.227
Light vegetable	.116	.010	**.646**	.016	-.108	-.014	.021	.014
Roots and stem vegetables	.094	.175	**.638**	-.053	.122	.028	.109	.017
Dark vegetables	.065	.169	**.478**	-.014	.227	-.081	-.059	-.072
Mushrooms	.160	.368	**.472**	-.001	.275	.043	.075	.130
Sea plants	.100	.274	**.415**	.018	.209	.077	.114	.252
Eggs	.290	.082	**.317**	.036	.007	.188	-.031	-.064
Liver	.071	.065	.021	**.708**	.059	-.019	.017	.027
Other viscus	.183	.073	-.044	**.691**	.047	.070	.039	.054
Other part of meat	.271	.092	-.033	**.513**	.021	.100	.073	.013
Animal blood	.237	.011	.007	**.414**	.035	.068	.065	.155
Melon	.088	.011	.144	-.012	**.679**	.107	-.029	.063
Bamboo	.169	.031	.064	.120	**.614**	.105	-.033	.168
Peacods	.050	.165	.311	.071	**.465**	-.026	.067	.085
Popsicle	.044	-.058	-.142	.040	.351	**.555**	.060	-.055
Ice cream	.082	.129	-.035	-.023	.116	**.553**	.163	-.064
Fried fast food	.122	.091	.058	.126	-.112	**.452**	-.030	-.010
Sweets/pudding	.043	.163	-.024	.030	.241	**.413**	.144	.103
Soy bean milk/jelly	.111	.311	.151	.001	-.025	**.408**	-.202	.187
Other fried food	.146	-.134	.186	.316	-.120	**.391**	.126	.059
Cookie	.071	.088	.056	.004	.075	.005	**.675**	-.026
Candy	.055	.073	.113	.133	-.126	.083	**.587**	.065
Cake	.131	.177	.101	.093	-.039	.217	**.412**	.078
Sweet preserved	.072	.179	-.142	.040	.138	.040	**.411**	.213
Fermented beans	.184	.207	-.035	.029	.166	-.043	.087	**.528**
Pickles	.166	-.023	.060	.202	.169	-.076	.123	**.480**
Other soy bean product	.019	.115	.385	-.021	.229	.066	.071	**.419**
Fired soy bean	-.015	.001	.276	.237	.018	.264	.155	**.343**
Milk powder	.091	.213	-.022	-.104	.205	-.026	.278	**-.338**

Note: Factor analysis: extracted by principle component analysis; rotated by Kaiser Varimax, explained variance =36.87%. Food categories: 1. animal protein; 2. whole grain fruits and dairy, 3. vegetables; 4. viscera and fats; 5.melon and bamboo; 6. Ice cream and fried food; 7. Sweets; 8. Pickle and others.
